# Lower Energy-Demanding Extraction of Bioactive Triterpene Acids by Microwave as the First Step towards Biorefining Residual Olive Skin

**DOI:** 10.3390/antiox13101212

**Published:** 2024-10-09

**Authors:** Irene Gómez-Cruz, María del Mar Contreras, Inmaculada Romero, Eulogio Castro

**Affiliations:** 1 Department of Chemical, Environmental and Materials Engineering, University of Jaén, Campus Las Lagunillas, 23071 Jaén, Spain; igcruz@ujaen.es (I.G.-C.); iromero@ujaen.es (I.R.); ecastro@ujaen.es (E.C.); 2 Institute of Biorefineries Research (I3B), University of Jaén, Campus Las Lagunillas, 23071 Jaén, Spain

**Keywords:** biorefinery, green extraction, maslinic acid, microwave-assisted extraction, oleanolic acid, olive pomace

## Abstract

In the olive oil industry, a pit fraction is collected from olive pomace and split into a clean pit fraction and a residual olive skin-rich fraction, which does not an industrial application. Therefore, in this work, microwave-assisted extraction (MAE) was applied to obtain high-value triterpene acids (maslinic acid and oleanolic acid) from this biomass using the renewable solvent ethanol. The response surface methodology was used to gain a deeper understanding of how the solvent (ethanol–water, 50–100% *v*/*v*), time (4–30 min), and temperature (50–120 °C) affect the extraction performance, as well as the energy required for the process. The effect of milling was also studied and the solid-to-liquid ratio was also evaluated, and overall, a good compromise was found at 10% (*w*/*v*) using the raw sample (unmilled biomass). The optimised conditions were applied to residual olive skin sourced from various industries, yielding up to 5.1 g/100 g and 2.2 g/100 g dry biomass for maslinic acid and oleanolic acid, respectively. In conclusion, the residual olive skin is a promising natural source of these triterpene acids, which can be extracted using MAE, releasing extracted solids rich in polymeric carbohydrates and lignin that can be valorised under a holistic biorefinery process.

## 1. Introduction

In recent years, the development of agro-industrial activities has led to an increase in the amount of waste generated and growing concerns about the environmental impacts they can have [1]. An alternative to preserve the environment and achieve an energy transition is to replace the current linear economy with a circular economy, where waste is reused and valorised with sustainability criteria [2,3].

Olive groves are the main crop in the Mediterranean basin. Its associated industries, such as olive oil mills and olive pomace oil extractors, generate numerous biomasses, some of which have limited industrial applications [3]. Their conversion into value-added products and energy through efficient and sustainable processes is crucial to contribute to the transition to carbon neutrality [4] and to support a green economy [5], rural development [6], and the achievement of the United Nations’ Sustainable Development Goals (SDGs) [7].

In particular, olive pomace (or alperujo) is the main byproduct of the two-phase olive oil extraction process, which contains pieces of stone, pulp, and skin. It is currently processed to obtain a partially destoned olive pomace, a pit fraction, and a residual fraction composed of mainly skin (residual olive skin or olive pomace skin) [3,8]. After destoning, olive pomace is extracted to obtain olive pomace oil, generating exhausted olive pomace as biomass. This biomass and the olive pits have applications as biofuels, but the residual olive skin has not been industrially exploited [3]. However, recent studies suggest that the residual olive skin could be used to obtain bioactive compounds, specifically triterpene acids (or triterpenic acids) [8]. Olive peels also contain phenolic compounds like hydroxytyrosol and tyrosol [9]. It should be noted that triterpene acids are mainly concentrated in fruit skin [10]; for example, maslinic acid and oleanolic acid have been described in the skin of olive fruits [11]. Both compounds have anti-inflammatory and antioxidant effects demonstrated in cells and in vivo, e.g., they can reduce the levels of reactive oxygen species (ROS) and nitric oxide (NO) [12,13,14]. Therefore, the residual olive skin has great potential for extracting these triterpenec acids, which have applications in the cosmetic, food, nutraceutical, pharmaceutical, and feed industries [12,13,14]. According to the literature, the technologies used for the extraction of triterpene acids from residual olive skin have been solid extraction with methanol/ethanol (1:1, *v*/*v*) for 5 min and centrifugation [8], Soxhlet extraction up to 60 min and 75 °C with ethyl acetate or methanol and microwave-assisted extraction (MAE) for 10 min at 85 °C with ethyl acetate or methanol [15]. In the latter study, MAE was the most efficient technology for the extraction of triterpene acids using ethyl acetate. However, the operational parameters affecting the extraction of these biocompounds have not been properly set for this biomass, particularly using ethanol. The use of ethanol has benefits compared to the use of other solvents, e.g., it can be produced from renewable sources. Another important issue is to find appropriate uses for the residual solid remaining after extraction, which is often ignored. Still, its valorisation is crucial to minimise waste and promote a more circular and eco-friendly economy.

In this line, the main objective novelty of this work was to evaluate microwave as an intensification technology to sustainably extract maslinic acid and oleanolic acid from the residual olive skin. For this purpose, the influence of crucial process parameters (milling, solvent, and microwave conditions) was evaluated and optimised to jointly maximise the extraction performance of these compounds and minimise energy consumption. Different samples were also extracted from different oil mills to capture the effect of this potential source of variability. Finally, the residual solids (extracted solids) were characterised to determine the impact of MAE on other components for subsequent conversion processes and integral biomass use.

## 2. Materials and Methods

### 2.1. Raw Material and Particle Size Fractionation

The residual olive skin was collected from a local company placed in Jaén (Spain) (Figure S1, sample 1). The moisture content was 4.9%. In the laboratory, a part of the sample was milled in an ultracentrifugal mill ZM 200 (Retsch, Haan, Germany) to a particle size of 1 mm.

In addition, a granulometric analysis of the raw biomass was carried out by sieving to determine the size of the different particles that make up this biomass. For this purpose, an analytical sieve shaker AS 200 control (Restch) was applied with sieves of several aperture sizes from 500 μm to 2 mm. All the fractions were weighted and then stored at room temperature.

For comparative purposes, samples of residual olive skin were provided by four different local industries in Jaén, Spain (Figure S1, samples 2 to 5). Additionally, olive pomace and exhausted olive pomace were acquired from olive industrial mills. All samples were dried at 45 °C in an oven (Memmert UF110, Schwabach, Germany) to a moisture lower than 10% *w*/*w* and stored in transparent polyethylene plastic bags with hermetic self-sealing, which isolated the biomass from the outside, and in the dark.

### 2.2. Reagents

Absolute ethanol was supplied from Honeywell (Morristown, NJ, USA). Acetonitrile, methanol, and orthophosphoric acid HPLC grade were from PanReac AppliChem (Barcelona, Spain). Ultrapure water was obtained using a Milli-Q system (Millipore, Bedford, MA, USA). Commercial standards of maslinic and oleanolic acids were purchased from Extrasynthese (Genay, France). The rest of the standards and reagents were obtained from Sigma-Aldrich (St. Louis, MO, USA).

### 2.3. Extraction of Triterpene Acids, Design of Experiments and Response Surface Methodology Application

#### 2.3.1. Microwave-Assisted Extraction

The extraction of triterpene acids was performed using a flexiWAVE microwave (Milestone Srl, Sorisole, Italy) with high-pressure vessels to avoid sample loss (limit of 100 bar). The temperature was kept under continuous control using a contactless infrared sensor during each extraction.

The residual olive skin and a milled portion (~1 mm) were each extracted following a Box–Behnken experimental design (BBD). The solid-to-liquid ratio was fixed at 10% (*w*/*v*) and the working volume was 30 mL. The BBD consisted of 17 experiments which were carried out independently per sample type and run in random order. In this design, the effect of three factors at three different experimental levels was evaluated: absolute ethanol percentage in water (50–100%, *v*/*v*), holding time (4–30 min), and holding temperature (50–120 °C). Each BBD included a central point at 75% *v*/*v* ethanol, 17 min, and 85 °C, respectively, which was tested five times for each BBD to help estimate the error in the analysis by the response surface methodology (RSM). Moreover, the responses analysed by RSM were the total extraction yield (or total extracted solids) (%, g/100 g dry biomass), the content of each triterpene acid (g/100 g dry biomass), and the energy consumed (kWh) in the extraction.

**Figure 1:**
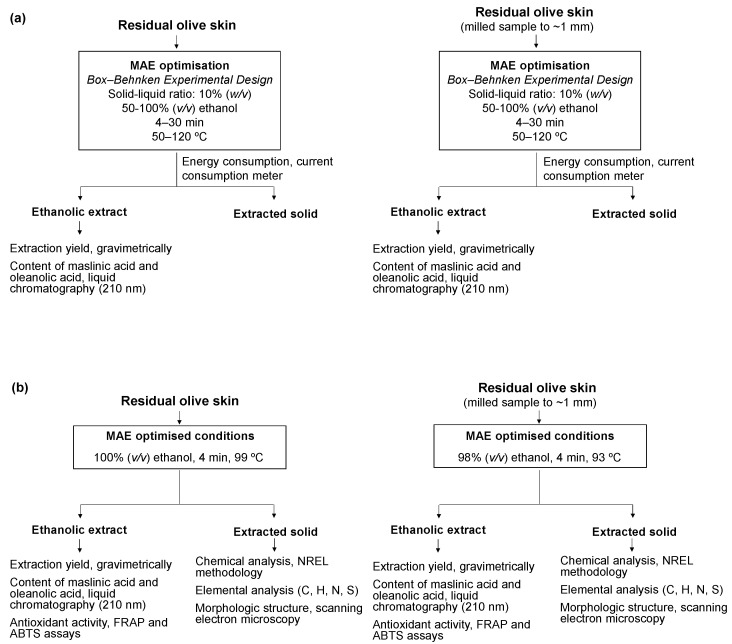
Scheme of the experimental procedure followed for the extraction of triterpene acids from residual olive skin by microwave-assisted extraction (MAE) and characterisation techniques: (**a**) for optimisation and (**b**) using optimised conditions. NREL, National Renewable Energy Laboratory.

After MAE, the samples were centrifuged (Herolab GmbH Laborgeräte, HiCen T, Wiesloch, Germany) for 10 min at 4000 rpm, obtaining a supernatant (an extract rich in triterpene acids) and a precipitate (extracted solid) (as an example, see Figure 1a). The extract was filtered through a nylon syringe filter (pore size 0.45 μm) (Grupo SinerLab, Madrid, Spain). A portion was used to determine the total extraction yield (Section 2.4) and another portion was analysed by high-performance liquid chromatography (HPLC) to determine the content of triterpene acids (Section 2.5.1.).

In all experiments, the energy consumption at the end of the experiments was measured using a current consumption meter (Gifort, Shanghai, China), according to a previous study [16]. The CO_2_ emission was estimated using a factor that 1 kWh equals 258 g of CO_2_ emission (the greenhouse gas emission intensity of electricity generation), considering the EU level stated by the European Environment Agency [17].

#### 2.3.2. Analysis of the Designs

The BBDs were analysed by RSM with Statgraphics Centurion 18 (Statgraphics Technologies, Inc., The Plains, VA, USA), which enabled us to obtain the statistical significance of the studied factors for each response, the mathematical models, and the response surfaces of the models. The models were evaluated using one-way analysis of variance (ANOVA) and the standardised Pareto plots for each factor were also obtained with the aforementioned software. The mathematical models were approximated by second-degree polynomial equations and refitted with those factors presenting significant regression coefficients at *p* < 0.10. Then, the coefficient of determination (R^2^), adjusted R^2^, the lack of fit, and the standard error of estimates were determined for the models. Once the models were created, the optimal extraction conditions were determined by the multiple-response optimisation of all the responses to maximise the overall desirability function, following the mathematical function described by previous studies [18,19]. The goal was to maximise the extraction yield and content of maslinic acid and olealonic acid and minimise energy consumption. The optimised extraction conditions (Figure 1b) were then tested (*n* = 10, each sample type) and the experimental data (extraction yield, the content of maslinic acid and oleanolic acid, and energy consumption) were compared to the predicted ones to validate the model. The relative error was measured as (experimental mean-predicted value) × 100/predicted value.

Once the optimal conditions were obtained, different solid-to-liquid ratios were tested (5–20%, *w*/*v*) using the raw biomass (that was unmilled) (sample 1, Figure S1) under optimised MAE conditions to evaluate how it affects the studied parameters. In addition, to evaluate the variability in the content of triterpene acids according to the production site, the optimised conditions were applied to samples acquired from four different local industries (Figure S1, samples 2 to 5).

#### 2.3.3. Solid–Liquid Extraction Assisted by Agitation

The extraction conditions were previously described in the work Gómez-Cruz et al. [20]. Briefly, it was performed using 100% ethanol at 25 °C for 24 h and 150 rpm in an orbital shaker (INFORS HT Ecotron, Thermo Fisher Scientific; Waltham, MA, USA).

### 2.4. Extraction Yield

The extraction yield was determined by drying 2 mL of extract at 105 °C for 24 h in the aforementioned oven to constant weight and weighting. The results were expressed as percentages, i.e., g extract/100 g biomass (on a dry basis). It also served to estimate the theoretical purity of the triterpenec acids, (content × 100)/extraction yield, which was expressed as g/100 dry extract.

### 2.5. Characterisation of the Extracts

#### 2.5.1. Characterisation of Triterpene Acids

The extracts were analysed by reversed-phase (RP)–high-performance liquid chromatography (HPLC) on a Shimadzu Prominence device (Kyoto, Japan) according to [20], and the column was HYPERSIL C18 BDS (250 mm × 4.6 mm; 5 µm particle size (Thermo Fisher Scientific Inc., Waltham, MA, USA) and a ternary solvent gradient was applied using 0.2% orthophosphoric acid aqueous solution, methanol, and acetonitrile at 1 mL/min at 30 °C [20]. The sample volume injected was 20 μL.

Calibration curves of commercial standards of maslinic acid and oleanolic acid were obtained at 210 nm by external standard calibration, according to a previous study [20]. The curves for maslinic acid and oleanolic acid were y = 8908x + 36,670 (R^2^ = 0.996) and y = 10,737x + 41,665 (R^2^ = 0.999), respectively. The results for both compounds were expressed as g/L or g/100 g biomass (on a dry basis).

#### 2.5.2. Antioxidant Activity

The antioxidant activity was determined by colourimetric assays using the ferric reducing power (FRAP) and ABTS™ radical scavenging assays according to Gómez-Cruz et al. [20]. Measurements were performed using the extracts obtained under optimised conditions, 593 nm and 734 nm, in 96-well plates, respectively, on a Bio-Rad iMark reader (Hercules, CA, USA) at room temperature. Briefly, in the former assay, the FRAP reagent was prepared by mixing 300 mM acetate buffer (pH 3.6), 10 mM 2,4,6-tris(2-pyridyl)-*s*-triazine (TPTZ) in 40 mM HCl, and 20 mM FeCl_3_ 6H_2_O, with a ratio of 10:1:1. Then, the diluted extracts/solvents (for blanks) were mixed with the FRAP reagent (100 μL into 3 mL) and reads were acquired after 6 min in the dark. In the second method, 2,2′-azinobis(3-ethylbenzothiazoline-6-sulfonate) (ABTS) at 7 mM was diluted with 2.45 mM K_2_S_2_O_8_ with phosphate buffer (pH 7.4) to an absorbance of 0.7. Then, this reagent (3 mL) was mixed with the diluted extract/solvents (for blanks) (30 μL) and the absorbance was measured after 6 min. Trolox was used as a standard to build calibration curves for ABTS (0–0.279 g/L) and FRAP (0–0.29 g/L). The results were expressed as g Trolox equivalents (TE)/100 g dry biomass.

### 2.6. Characterisation of the Solids Resulting from MAE

#### 2.6.1. Chemical Characterisation

Figure 1b summarises the methodology and analyses applied for residual olive skin and the extracted solids recovered after MAE under optimised conditions of unmilled and milled sample 1. The chemical composition of these samples was determined according to the methodology described by the National Renewable Energy Laboratory (NREL) [20]. Briefly, this methodology was used to determine the moisture and the content of ash at 105 °C and 575 °C, respectively, and the content of extractives (extractable components) by Soxhlet extraction with water and ethanol, while polymeric carbohydrates and lignin were estimated after acid hydrolysis using HPLC with refractive index detection to quantify the monomeric sugars (arabinose, galactose, glucose, mannose, and xylose) and gravimetric analysis, respectively.

In addition, a TruSpec Micro device (Leco, St. Joseph, MI, USA) was employed to analyse the content of carbon, hydrogen, nitrogen, and sulphur through combustion and an analysis of the generated gases using infrared detection (CO_2_, H_2_O, and SO_2_) and thermal conductivity detection (N_2_). The nitrogen content was used to estimate the protein content using the conversion factor 6.25 [21].

#### 2.6.2. Scanning Electron Microscopy

The raw biomass and the extracted solids were analysed using field emission scanning electron microscopy (SEM) (Merlin Carl Zeiss equipment) (Carl Zeiss, Oberkochen, Germany). The dried samples were metallised with gold and bombarded with a field emission electron source to obtain high-resolution images at 1000× and 64× magnification, i.e., scales of 50 μm and 500 μm, respectively.

### 2.7. Statistical Analysis

The BBD designs were evaluated as commented in Section 2.3.2. The software Statgraphics Centurion 18 was also applied to carry out an F-test and *t*-test for the comparison of the experimental results obtained by reproducing the optimised conditions using unmilled (raw) and milled residual olive skin. To compare the data in the other tables, a ANOVA was conducted followed by Fisher’s least significant difference (LSD) test. The significance level was set at *p* < 0.05.

## 3. Results

### 3.1. Raw Biomass Characterisation and Particle-Size Distribution

The current exploitation of olive pomace pits as a biofuel has led to the generation of a residual olive skin-rich biomass (residual olive skin), a poorly explored waste that deserves applications to minimise waste associated with the olive oil industry. Therefore, this bioresource (sample 1) was chemically characterised (Table 1, second column). The results showed that this biomass is mainly composed of about 35% (*w*/*w*) lignin, 24% (*w*/*w*) extractives (extractable components), and 25% (*w*/*w*) structural carbohydrates. The latter were composed of cellulose (as glucose) and hemicellulose (mainly, xylans) with a similar percentage, 12.4% and 13.1%, respectively. Compared with other olive biomasses, these data suggest that its lignin content is similar to that of olive pits [22] and higher than that of olive pomace and exhausted olive pomace [20,22,23]. The polysaccharide content is lower than in olive pits and slightly higher than in olive pomace and exhausted olive pomace [20,22,23,24].

This bioresource contained a high content of ethanolic extractives (16.3%). It is higher than that of olive pits [22] and exhausted olive pomace [20,22] but in the range of olive pomace [23]. Ethanolic extractives may contain pigments, waxes, and other cuticle components of olive skin [24]. Waxes contain bioactive compounds, like triterpenoids, which are polycyclic hydrocarbons with high potential to be used in drugs, functional foods, and healthcare products [25]. Particularly, in the olive fruit, pentacyclic triterpene acids, a type of triterpenoid, are major components of the wax, being present in the skin [26]. Since olive oil contains low quantities of these biocompounds, they could pass to the olive pomace [25], being concentrated in the residual olive skin after the separation of the olive pits [8]. Therefore, this biomass was used as a feedstock to obtain high-value triterpene acids in the following section.

Concerning the particle distribution, the residual olive skin (as received) is sorted by sieving since it is an important factor that can impact the extraction performance. It was composed of particles with a size higher than 2 mm (F1) (7.55%, *w*/*w*), 1–2 mm (F2) (38.65%, *w*/*w*), 0.85–1 mm (F3) (10.26%, *w*/*w*), 0.5–1 mm (F4) (27.29%, *w*/*w*), and <0.5 mm (the bottom of the sieve) (F5) (16.26%) (Figure S2). The coarse fraction (F1) was rich in pits (see the images in Figure S2). A part of the pits can be strained into the residual olive skin after the cleaning process. It has been suggested that pits are very resistant to rupture and present higher diameters than those particles from the skin and flesh. Alternatively, the latter fractions are more easily broken and have smaller diameters [27].

### 3.2. Evaluation of the Extraction of Triterpene Acids by MAE

#### 3.2.1. Influence of Operating MAE Parameters on Unmilled and Milled Samples

Our preliminary studies have shown that using absolute ethanol, MAE (holding temperature 120 °C and holding time 4 min) showed higher efficiency for extracting triterpene acids than conventional solid–liquid extraction with agitation (24 h and 150 rpm), achieving nearly 17% higher content. Therefore, in this work, MAE was applied to extract triterpene acids considering energy aspects to minimise the carbon footprint associated with this process. Temperature, which is a crucial parameter in extraction [28], was evaluated from 50 to 120 °C, according to the preliminary study. The other studied factor was the holding time required to extract the target compounds in the range from 4 to 30 min. Ethanol was selected as a solvent for MAE since it can be obtained from renewable resources and it was a suitable option to recover triterpene acids according to previous studies on other types of olive biomasses [16,28]. This solvent also has a high capacity to absorb microwave energy and transfer it to the biomass being extracted due to its high dielectric constant (24.3) and dielectric loss (22.9), respectively, which is desirable when using microwaves [29]. However, studies on other biomasses tested aqueous–alcoholic solutions for extracting triterpene acids [3], and thus, the ethanol percentage was evaluated in the BBD.

Two BBDs were then tested, one using the raw residual olive skin and the other with a milled portion of the biomass to assess the effect of further milling (with a particle size ≤ 1 mm). Table 2 details the experimental data obtained for both types of samples at a solid-to-liquid ratio of 10% (*w*/*v*). The results were similar in both designs, being slightly higher using the milled samples for some experiments. For example, for the unmilled sample, the values were 5.84–20.79% (extraction yield), 0.25–2.46 g/100 g (maslinic acid), and 0.03–1.07 g/100 g (oleanolic acid) dry biomass. For the milled sample, the values varied as follows: 5.98–20.05% (extraction yield), 0.36–2.64 g/100 g (maslinic acid), and 0.01–1.10 g/100 g (oleanolic acid) dry biomass.

**Figure 2:**
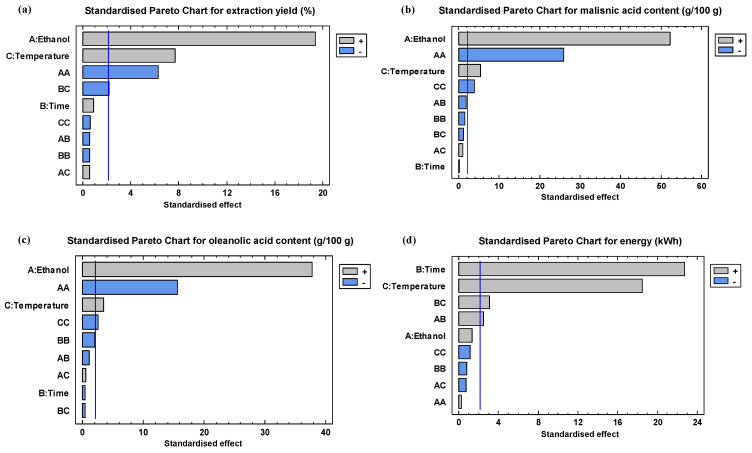
Effect of the factors in the response variables represented in Pareto charts: (**a**) extraction yield (%), (**b**) maslinic acid content (g/100 g), (**c**) oleanolic acid content (g/100 g), and (**d**) energy consumption (kWh) for the unmilled biomass. In each chart, the vertical blue line indicates the significance of the effects at 90% confidence level.

**Figure 3:**
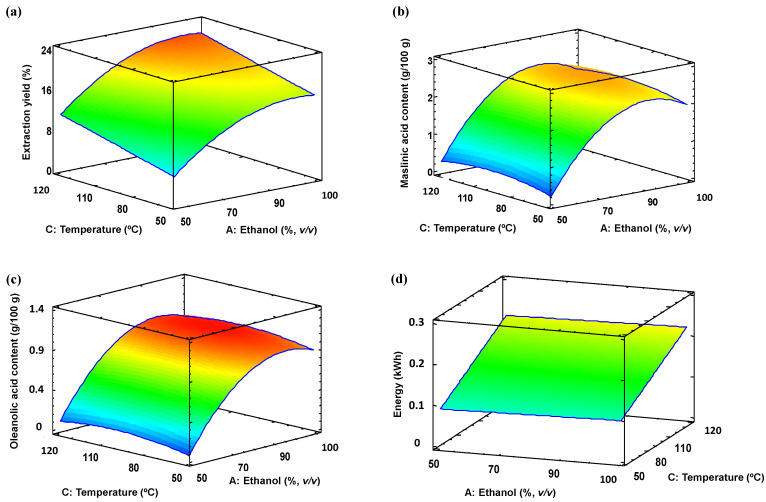
Effect of the factors in the response variables represented in the response surface charts as a function of ethanol percentage and extraction temperature: (**a**) extraction yield (%), (**b**) maslinic acid content (g/100 g), (**c**) oleanolic acid content (g/100 g), and (**d**) energy consumption (kWh) for the unmilled biomass. The holding time was fixed at 17 min. The colour gradient from blue to red indicates low to high values for a response variable.

In both cases, the main effects of the independent variables and their interactions on the studied responses were evaluated using Pareto charts (Figure 2) and response surfaces (Figure 3). As shown in Figure 2a, Figure 3a and Figure S3a, the ethanol percentage (linear term) was the process variable that most influenced the extraction yield and positively, followed by temperature (linear term), regardless of whether the biomass was milled or not. The former parameter also significantly influenced the content of both triterpene acids (Figure 2b,c, Figure 3b,c and Figure S3b,c). In all cases, owing to the negative influence of the quadratic term of ethanol percentage, a maximum in the solubilisation is achieved between 80 and 100% ethanol (Figure 3a–c and Figure S3a–c). It has been observed that when MAE was employed on olive pomace pulverised at 0.35 mm the ethanol percentage up to 90% improved the extraction yield of triterpene acids [16]. The effect of the holding temperature was also significant for these responses when the unmilled (raw) biomass was used (Figure 2b,c and Figure 3b,c) This may be because, at higher temperatures, the viscosity of the solvent is reduced, allowing it to penetrate deeper into the biomass, thus increasing the solubility of desired compounds [30]. Concerning the holding time, it only influenced the extraction yield, suggesting that triterpene acids were quickly solubilised.

In the case of energy consumption, the linear term of the time followed by temperature were the most significant factors in MAE (Figure 2d and Figure S3d). Therefore, these results suggest that a short holding time can be applied to extract triterpene acids from residual olive skin with minimal effect on their extraction performance, while it would significantly diminish the energy consumption of the process and hence the CO_2_ emission. In this line, MAE offers a high mass transfer of triterpene acids to the polar organic solvent at medium to moderately high temperatures (50–80 °C) in a short time (4–6 min) as previous studies have shown using olive skin [15] and olive pomace [16].

#### 3.2.2. Model Fitting and Multiple Optimisation

For both experimental designs, a multiple regression fit was applied to obtain the second-degree polynomial equations (models) describing the relationship between each response and the three independent variables whenever the effect was significant (Table 3). The statistical analysis of the models is summarised in Table 3. The models fitted well (*p* < 0.0001) and the adjusted coefficients of determination (R^2^ adj) were in the range from 0.9764 to 0.9907 for the unmilled residual olive skin and between 0.9704 and 0.9852 for the milled biomass, suggesting that the experimental data matched well with the predicted values. Furthermore, the coefficient values (CVs) were less than 10% in both cases, suggesting adequate dispersion to explain the relationship between the operating factors and the different responses.

Once the individual responses were modelled, to design a sustainable extraction process, the strategy consisted of simultaneously maximising the extraction yield (%) and the content of the triterpene acids (g/100 g) while minimising the energy consumption (kWh). For this purpose, multiple-response optimisation was applied by obtaining the desirability function for the unmilled and milled residual olive skin. This mathematical tool could help find a good compromise for process designing because improving only one response can worsen the other ones [18]. That is, it is important to maximise triterpene acid extraction considering the energy input, which is crucial to reduce the production costs associated with this process. For example, the energy consumption varied in the BBD experiments between 0.045 kWh (11.6 CO_2_ equivalents) and 0.256 kWh (66.0 CO_2_ equivalents), the latter being nearly six times higher.

Table 4 shows the optimal conditions and data predicted for each sample type with the desirability function of 0.9204 and 0.8637, respectively, giving equal weightage for all responses (Figure S4). This means that the optimality predicted by the model is adequate, i.e., the closer this value is to 1, the better the simultaneous optimisation [18]. Moreover, the best desirability values were in a similar range in both cases as the surface plots show. The optimal conditions predicted were very close (Table 4). In this sense, Table 4 highlights that 99 °C with 100% (*w*/*v*) ethanol and 93 °C (*w*/*v*) with 98% ethanol for 4 min are adequate to extract triterpene acids when the biomass is used as itself (unmilled) and milled, respectively. These conditions were tested and the experimental results confirmed the suitability of the models, i.e., most errors were lower than 10% when compared with the theoretical values.

Although the reduction in the size can be suitable to increase the transfer of bioactive compounds from biomass [31], in this case, a large part of the particle size of the raw biomass was lower than 1 mm (i.e., the sum of F3, F4, and F5 was 53.9%, *w*/*w*) (Figure S2). This is advantageous since it can explain that only a slight increase in the holding temperature (6 °C) was required for this type of sample compared with the milled one. Experimentally, this implied slightly more, but not significant, energy consumption (0.097 vs. 0.093 kWh), i.e., 0.004 kWh (*p* = 0.057). Moreover, the values were very close for both biomasses (unmilled and milled samples) used in the designs, but with significant differences (*p*-values between 0.002 and 0.045). Overall, using both sample types, it can be extracted at optimal conditions about 3.45 and 3.51 g/100 g of triterpene acids, with 70% being maslinic acid. This agrees with the results found by Romero et al. [8] and Fernández-Pastor et al. [15] who obtained a similar content and ratio of the maslinic acid value when olive skin milled (<250 μm) and unmilled was used with ethyl acetate or methanol. This suggests that the milling operation step can be saved and reduce the economic and energetic cost of the process; at the lab scale, the energy cost is about 0.1 kWh/kg biomass.

Moreover, the use of ethanol as a solvent is attractive compared to other solvents applied in olive biomasses, e.g., hexane [32], methanol [15], and ethyl acetate [15], considering safety, health, and environmental aspects [33]. It is also an excellent volatile organic solvent, which can be obtained using renewable resources and purified by distillation to be reutilised, overall reducing the carbon footprint of the extraction process [34].

The antioxidant activity (FRAP and ABTS assays) of the ethanolic extracts was also evaluated under optimised conditions (Table 4). The data obtained are similar for both types of extracts. Previous studies have shown that oleanolic acid and other triterpene acids are proton donors, with the ability to scavenge NO^•^ and ^•^O^2−^ radicals and prevent lipid peroxidation [35]. Moreover, the antioxidant role of the studied triterpene acids has been assessed in cells and in vivo, being able to reduce the levels of ROS and NO [12,13,14]. A recent study suggests, for example, that maslinic acid can prevent oxidative stress and modulate inducible nitric oxide synthase (iNOS), which has been associated with an anti-inflammatory effect in mice treated with lipopolysaccharide [36]. Oleanolic acid can also mitigate oxidative stress, with the potential to target diabetes, improving insulin signalling and sensitivity with better glucose homeostasis, among other effects [14].

### 3.3. Evaluation of the Solid-to-Liquid Ratio in the Extraction of Triterpene Acids

**Figure 4:**
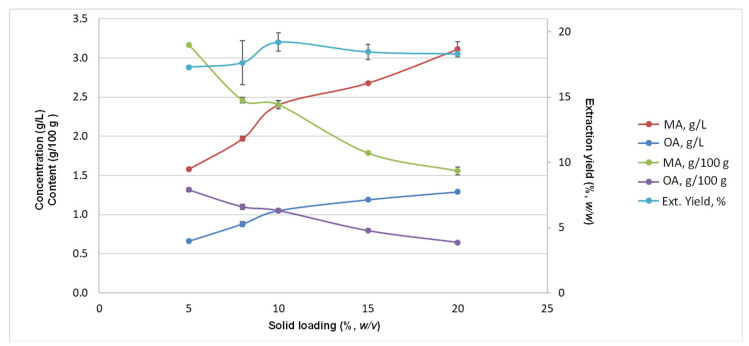
Influence of the solid-to-liquid ratio on the total extraction (ext.) yield (%), the concentration of maslinic acid (MA) and oleanolic acid (OA) (g/L) in the ethanolic extract and their content expressed as g/100 g of biomass (on a dry basis).

Given the similar results obtained for the aforementioned designs with unmilled (raw) and milled samples (Section 3.2), the milling stage can be saved to reduce operations in the overall process. The starting biomass (unmilled) was thus selected to evaluate the extraction of triterpene acids with different solid-to-liquid ratios under the optimised conditions (100% ethanol and 99 °C for 4 min). In Figure 4, it is observed that although the concentration of these compounds increases using a higher solid-to-liquid ratio, the extraction per gram of biomass presents a different trend; that is, although the extracted liquid fraction is concentrated in these compounds, a part of them remains in the solid fraction after extraction. This may be because triterpene acids present sufficient solvency, but the mass transfer is not completed at a high solid-to-liquid ratio [37].

Therefore, three ways can be followed considering these results (Figure 4): (a) One approach is to use a high solid-to-liquid ratio, e.g., 20% *w*/*v*, to industrially work with concentrated liquid extracts (total, 4.4 g/L). The theoretical purity of the solid extract could be about 11% (*w*/*w*) as a dry extract. (b) The second is to use a low solid-to-liquid ratio (~5% *w*/*v*) to obtain more solubilisation of triterpene acids per gram of biomass, a lower concentration (2.2 g/L), and dry rich extracts in triterpene acids (theoretical purity ~25% *w*/*w*, dry extract). (c) The third is to use intermediate solid-to-liquid ratios (~10% *w*/*v*) to obtain high yields, intermediate concentration and content (3.4 g/L; 3.45 g/100 g biomass), and rich extracts with a purity of about 17% *w*/*w*, dry extract. Concerning the energy consumption, a slight increase from 5% (*w*/*v*) (0.089 kWh) to 20% (*w*/*v*) (0.100 kWh) was observed, with an intermediate value at 10% (*w*/*v*) (0.093 kWh). It has been suggested that using low solid-to-liquid ratios allows biomass particles to absorb more microwave energy per gram. It can be related to both a reduced energy consumption and a high solubilisation of the bioactive compounds to the surrounding solvent [38].

Overall, for the large obtainment of triterpene acids, the application of a solid-to-liquid ratio of 10% (*w*/*v*) (option c) could be a good compromise option. Compared to option a, it offers to work with double biomass per volume of solvent, reducing solvent consumption and production costs. The energy cost per g of triterpene acids will also be intermediate between the other options. Alternatively, the other options are also valid, depending on the industrial purposes.

### 3.4. Extraction of Triterpene Acids from the Residual Olive Skin of Different Origins and Related Biomasses

The content of triterpenoids is affected by the fruit ripening and the fruit type [26,39,40], and hence, the content of this type of compound in the residual olive skin may vary with the seasons and from mill to mill. In the present work, besides the studied residual olive skin sample, four other samples (samples 2 to 5) (Figure S1) were collected from four local industries in the 2022/2023 harvesting season, dried and extracted using the optimised MAE conditions for unmilled biomass (100% ethanol, 4 min, and 99 °C) for comparison. Moreover, samples from olive pomace and exhausted olive pomace were also extracted. Table 5 shows the values of the extraction yield (%), the content of maslinic acid and oleanolic acid (g/100 g), and the energy consumption (kWh) for these samples.

The variation in the concentration of triterpene acids was notable (3.15–5.13 g/100 g), although all samples contained about 70% maslinic acid. Potential factors affecting the content of triterpene acids in residual olive skin are agronomical factors (e.g., olive cultivar and maturity of the fruit), as commented before, and technological ones, e.g., the extraction process of olive oil [3]. A large variation in the content of triterpene acids was also found in this biomass by other authors [8], but in this case, the moisture content, varying from 7 to 50%, could also affect the extraction.

### 3.5. Characterisation of the Extracted Solids

The extracted solids resulting from the extraction process of marketable natural biocompounds (bioactive compounds, colourants, essential oils, etc.) are new wastes and their exploitation is crucial to meet circularity. Therefore, the extracted solids recovered after MAE were further characterised (as Figure 1b shows) as a case of study and compared to the starting biomass (sample 1) to obtain an insight into how this step affects other components from the residual olive skin.

The chemical characterisation is shown in Table 1 for the extracted solids from unmilled (raw) and milled residual olive skin after MAE. As expected, MAE with ethanol removed mainly extractable components due to the polarity affinity of these extractives with the extraction solvent. In turn, this increased the percentage of lignin in the extracted solids. MAE barely affected the glucan content but solubilised about 17% of hemicellulosic sugars, mainly xylans. Xylans are more susceptible to solubilisation than cellulose and lignin when utilising pretreatments based on ethanolic solutions (organosolv pretreatments) to break down olive biomass. The solubilisation degree depends on the temperature, ethanol concentration, and whether a catalyst is added [41]. However, overall, the extracted solids still contained about 23% (*w*/*w*, on a dry basis) of polymeric carbohydrates. These components can be deconstructed into their monomeric sugars through a subsequent stronger pretreatment and then fermented to generate biofuels or build block chemicals in a biorefinery based on the residual olive skin [42]. The content of lignin was closer to 40% (*w*/*w*, on a dry basis), which could be interesting for producing aromatic compounds [43] and for the formulation of various bioproducts, e.g., biochar [44].

Regarding the ultimate composition of the extracted solids and compared to the raw biomass, MAE caused a decrease in the carbon and hydrogen contents, while oxygen was increased (Table 1). Wax components are hydrocarbons without oxygen or with a low content of this element (e.g., triterpenoids) [33], and so their extraction will reduce the ratio of the former elements to oxygen in the resulting extracted solid.

Moreover, the raw residual olive skin (unmilled) and the extracted solid obtained after MAE (optimised conditions) were evaluated by SEM. Figure S5a,b show SEM images of them, respectively. At a higher magnification, Figure S5a2,b2,a3,b3 show the epicarp surface with flanges and epidermal cells of the olive fruit tissue in the latter biomasses, respectively, according to a previous study [45]. These structures were more evident in this biomass than in exhausted olive pomace [20], suggesting again that the studied biomass was enriched in the skin part. It was also evidenced that the different structures presented in the residual olive skin (Figure S5a1) were more fractured after MAE (Figure S5b1). The action of microwave penetration into the material may cause intracellular pressure and the destruction of the cellular structures, which increases the permeability of biomass, generating transfer channels that contribute to intensifying the extraction [46,47]. Microwave energy can raise the temperature to evaporate the ethanol inside cells until the pressure exceeds the yield strength to rupture the cell wall and cause these channels [48].

It has been suggested that the extraction of wax components, such as triterpenoids, can impact not only the composition and structure of the biomass but also the downstream processing. For example, the supercritical fluid extraction of waxes from maize stover favoured the obtainment of biofuels [49]. Therefore, further studies should be performed to evaluate the integration of the MAE step to obtain triterpene acids in a biorefinery cascading scheme based on the residual olive skin.

## 4. Conclusions

Residual olive skin can be used as a raw material to obtain bioactive triterpene acids without the need for prior milling, thus saving costs. Using multiple optimisation, the optimised extraction conditions were 100% *v*/*v* ethanol at 99 °C for 4 min, allowing the energy-efficient extraction of maslinic acid and oleanolic acid. These extraction conditions were applied to samples obtained in several olive oil mills and the content of maslinic acid varied between 2.40 and 5.13 g/100 g, while oleanolic acid ranged from 1.05 to 2.19 g/100 g. The chemical composition of the residual solids showed the presence of polymeric carbohydrates and lignin as the main components, which can be valorised after MAE under a holistic biorefinery process, meeting the circular bioeconomy and energy transition targets. Overall, these results could be useful for the future large obtainment of these triterpene acids from residual olive skin using MAE.

## Figures and Tables

**Table 1:** Chemical and elemental composition of raw residual olive skin and extracted solids obtained under optimised conditions by microwave-assisted extraction. Data (%, dry weight basis) represent the mean value and standard deviation (*n* = 3).

Component	Raw Biomass	Extracted Solid from Unmilled Biomass	Extracted Solid from Milled Biomass
Chemical characterisation	%	%	%
Extractives	23.80 ± 0.34 ^a^	13.36 ± 0.91 ^b^	14.26 ± 0.31 ^b^
Aqueous extractives	7.49 ± 0.33 ^a^	5.28 ± 0.78 ^b^	4.67 ± 0.14 ^b^
Ethanol extractives	16.31 ± 0.08 ^a^	8.08 ± 0.17 ^c^	9.59 ± 0.18 ^b^
Cellulose (as glucose)	12.39 ± 2.03 ^a^	12.13 ± 0.77 ^a^	12.60 ± 0.54 ^a^
Hemicellulose	13.07 ± 2.23 ^a^	10.89 ± 0.64 ^a^	10.89 ± 0.77 ^a^
Xylan	12.48 ± 2.09 ^a^	10.06 ± 0.64 ^a^	10.35 ± 0.77 ^a^
Galactan	0.32 ± 0.04 ^a^	0.63 ± 0.06 ^b^	0.39 ± 0.03 ^a^
Arabinan	0.27 ± 0.11 ^a^	0.23 ± 0.04 ^a^	0.15 ± 0.03 ^a^
Acetyl groups	1.76 ± 0.25 ^a^	1.58 ± 0.15 ^a^	1.56 ± 0.17 ^a^
Lignin	34.41 ± 0.99 ^b^	40.51 ± 1.45 ^a^	38.07 ± 0.80 ^a^
Acid insoluble lignin	33.17 ± 1.17 ^a^	39.59 ± 1.44 ^a^	37.81 ± 1.15 ^a^
Acid soluble lignin	1.24 ± 0.19 ^a^	0.92 ± 0.13 ^b^	0.84 ± 0.01 ^b^
Protein	3.95 ± 0.23 ^a^	3.27 ± 0.73 ^a^	3.79 ± 0.47 ^a^
Ash	0.42 ± 0.07 ^ab^	0.34 ± 0.01 ^b^	0. 50 ± 0.03 ^a^
Ultimate analysis ^1^	%	%	%
Carbon	57.58 ± 1.17 ^a^	50.01 ± 0.72 ^b^	51.96 ± 1.38 ^b^
Hydrogen	7.81 ± 0.26 ^a^	6.62 ± 0.17 ^b^	6.81 ± 0.15 ^b^
Nitrogen	0.63 ± 0.04 ^a^	0.52 ± 0.12 ^a^	0.61 ± 0.08 ^a^
Sulphur	0.33 ± 0.13 ^a^	0.12 ± 0.09 ^a^	0.17 ± 0.09 ^a^

^1^ The content of oxygen is 31.95% (raw biomass), 41.38% (extracted solid from unmilled biomass), and 39.13% (extracted solid from milled biomass), obtained by difference: 100-N%-C%-H%-S%-ash%. Different letters indicate statistically significant differences between means (*p* < 0.05).

**Table 2:** Box–Behnken experiments and results obtained using unmilled and milled residual olive skin for the response variables: energy consumption, extraction yield, and content of maslinic acid and oleanolic acid. Coded values for factors are shown within parentheses.

Run	Ethanol (%, *v*/*v*)	Time (min)	Temperature	Energy Consumed ^1^	Ext. Yield (%, *w*/*w*)		Maslinic Acid (g/100 g)		Oleanolic Acid (g/100 g)	
			(°C)	(kWh)	Unmilled	Milled	Unmilled	Milled	Unmilled	Milled
1 *	75 (0)	17 (0)	85 (0)	0.140	16.11	15.21	2.18	2.46	0.89	0.96
2	100 (1)	17 (0)	120 (1)	0.191	20.79	20.05	2.46	2.64	1.05	1.10
3	50 (−1)	17 (0)	50 (−1)	0.087	5.84	5.98	0.25	0.37	0.03	0.05
4	100 (1)	4 (−1)	85 (0)	0.077	19.00	16.01	2.45	2.48	1.07	1.04
5	75 (0)	30 (1)	50 (−1)	0.137	14.52	11.6	1.87	2.08	0.70	0.79
6	50 (−1)	17 (0)	120 (1)	0.197	9.42	10.03	0.29	0.26	0.03	0.02
7	75 (0)	4 (−1)	50 (−1)	0.045	11.46	10.65	1.78	2.00	0.69	0.78
8	75 (0)	30 (1)	120 (1)	0.254	17.46	17.51	2.13	2.46	0.85	0.94
9	100 (1)	17 (0)	50 (−1)	0.092	16.34	16.3	2.31	2.51	1.01	1.07
10 *	75 (0)	17 (0)	85 (0)	0.148	16.75	14.45	2.04	2.04	0.80	0.83
11 *	75 (0)	17 (0)	85 (0)	0.157	14.92	14.76	2.17	2.07	0.89	0.86
12	100 (1)	30 (1)	85(0)	0.224	18.19	18.57	2.31	2.34	1.01	1.01
13	75 (0)	4 (−1)	120 (1)	0.115	17.84	15.68	2.16	2.14	0.87	0.89
14	50 (−1)	4 (−1)	85 (0)	0.081	7.61	7.61	0.37	0.36	0.03	0.03
15 *	75 (0)	17 (0)	85 (0)	0.139	14.99	14.71	2.13	2.07	0.87	0.85
16	50 (−1)	30 (1)	85 (0)	0.190	7.71	7.95	0.44	0.42	0.05	0.01
17 *	75 (0)	17 (0)	85 (0)	0.141	16.13	15.28	2.14	2.11	0.85	0.87

* Central points of the experimental design. ^1^ Total energy consumption of the two-sample extraction process (kWh).

**Table 3:** Mathematical models obtained in the Box–Behnken designs using coded values for each response variable and statistical results.

Studied Parameter	Models Equations	CV (%)	R^2^	Adjusted R^2^	F-Value ^1^	Lack of Fit (*p*-Value)
Unmilled residual olive skin						
Extraction yield (%)	15.57 + 5.47∙A + 0.25∙B + 2.17∙C − 0.86∙B∙C − 2.46∙A^2^ (1)	5.5	0.9838	0.9764	0.76	0.50
Maslinic acid (g/100 g)	2.11 + 1.02∙A + 0.10∙C − 0.70∙A^2^ − 0.11∙A^2^ (2)	3.5	0.9931	0.9907	3.12	0.08
Oleanolic acid (g/100 g)	0.84 + 0.50∙A + 0.04∙C − 0.29∙A^2^ − 0.05∙C^2^ (3)	4.6	0.9899	0.9865	4.19	0.05
Consumed energy (kWh)	0.14 + 0.004∙A + 0.061∙B + 0.049∙C + 0.009∙A∙B − 0.012∙B∙C (4)	5.3	0.9874	0.9817	1.02	0.52
Milled residual olive skin						
Extraction yield (%)	14.78 + 4.92∙A + 0.71∙B + 2.34∙C + 0.55∙A∙B– 1.57∙A^2^ − 0.80∙B^2^ (5)	2.6	0.9908	0.9852	2.55	0.19
Maslinic acid (g/100 g)	2.16 + 1.07∙A − 0.74∙A^2^ (6)	8.1	0.9741	0.9704	-	-
Oleanolic acid (g/100 g)	0.86 + 0.51∙A − 0.32∙A^2^ (7)	7.0	0.9865	0.9845	-	-
Consumed energy (kWh)	0.14 + 0.004∙A + 0.061∙B + 0.049∙C − 0.003∙B^2^ − 0.012∙B∙C (8)	5.3	0.9874	0.9817	1.02	0.52

^1^ *p*-value < 0.05—cannot be determined because there are not enough degrees of freedom. A: ethanol concentration; B: time; C: temperature; CV, coefficient of variation. CV was estimated as the standard error of estimates (SEEs) × 100/mean.

**Table 4:** Predicted results by the model at the optimised conditions and experimental results measured after the application of these conditions in the raw (unmilled) and milled residual olive skin. The antioxidant activity (FRAP and ABTS) of the extracts is also provided. Data represent the mean value and standard deviation (*n* = 10).

Studied Parameter	Unmilled Residual Olive Skin			Milled Residual Olive Skin		
Optimal conditions and desirability value	100% ethanol, 4 min, 99 °C (0.9204) ^1^			98% ethanol, 4 min, 93 °C (0.8637) ^1^		
Studied parameter	Predicted values	Experimental values	Error (%)	Predicted values	Experimental values	Error (%)
Extraction yield (%)	19.50	19.21 ± 0.71 ^a^	1.4	16.48	18.55 ± 0.73 ^a^	11.1
Maslinic acid (g/100 g)	2.46	2.40 ± 0.05 ^a^	2.5	2.52	2.43 ± 0.05 ^a^	3.7
Oleanolic acid (g/100 g)	1.07	1.05 ± 0.02 ^b^	1.9	1.06	1.07 ± 0.02 ^a^	0.9
FRAP (g TE/100 g)	-	0.70 ± 0.04 ^b^	-	-	0.82 ± 0.03 ^a^	-
ABTS (g TE/100 g)	-	1.80 ± 0.20 ^a^	-	-	1.91 ± 0.18 ^a^	-
Consumed energy (kWh)	0.090	0.097 ± 0.001 ^a^	7.5	0.085	0.093 ± 0.001 ^a^	9.7
Carbon emission (g CO_2_ equivalent)	-	25.03 ± 0.26 ^a^	-	-	23.99 ± 0.26 ^a^	-

GAE: gallic acid equivalent; TE: Trolox equivalent. ^1^ Desirability function value in brackets. Different letters indicate statistically significant differences between means (*p* < 0.05).

**Table 5:** Extraction yield, content of maslinic acid and oleanolic acid and energy consumption of different samples of residual olive skin, olive pomace and exhausted olive pomace.

Samples	Extraction Yield (%, *w*/*w*)	Maslinic Acid (g/100 g)	Oleanolic Acid (g/100 g)	Consumed Energy ^1^ (kWh)
Different residual olive skin samples				
Sample 1	19.21 ± 0.71 ^e^	2.40 ± 0.05 ^e^	1.05 ± 0.02 ^e^	0.097
Sample 2	25.07 ± 0.64 ^b^	4.21 ± 0.04 ^b^	1.56 ± 0.01 ^b^	0.092
Sample 3	17.69 ± 0.33 ^f^	3.15 ± 0.00 ^d^	1.30 ± 0.01 ^d^	0.091
Sample 4	33.02 ± 0.66 ^a^	5.13 ± 0.00 ^a^	2.19 ± 0.00 ^a^	0.092
Sample 5	21.05 ± 0.27 ^d^	3.50 ± 0.06 ^c^	1.45 ± 0.01 ^c^	0.090
Other biomasses				
Olive pomace	22.70 ± 0.95 ^c^	0.54 ± 0.05 ^f^	0.19 ± 0.02 ^f^	N.D.
Exhausted olive pomace	15.23 ± 1.57 ^g^	0.53 ± 0.04 ^f^	0.19 ± 0.01 ^f^	N.D.

N.D.: not determined; ^1^ total energy consumption of the two-sample extraction process (kWh) carried out once. Different letters indicate statistically significant differences between means (*p* < 0.05).

## Data Availability

The original contributions presented in the study are included in the article/supplementary material, further inquiries can be directed to the corresponding author/s.

## Supplementary Materials

The following supporting information can be downloaded at https://www.mdpi.com/article/10.3390/antiox13101212/s1, Figure S1: Images of the different residual olive skin samples and geographic coordinates of the local industries; Figure S2: Images of residual olive skin after sieving and weight distribution; Figure S3: Effect of the independent factors in the response variables represented in Pareto charts (left) and response surface charts (right) as a function of ethanol percentage and extraction temperature: (a) extraction yield (%), (b) maslinic acid content (g/100 g), (c) oleanolic acid content (g/100 g), and (d) energy consumption (kWh) for the milled biomass. The holding time was fixed at 17 min; Figure S4: Three-dimensional plots of the overall desirability response surface for the effects of ethanol concentration and temperature in (a) microwave-assisted extraction of the raw (unmilled) and (b) milled residual olive skin. The time was fixed at 17 min; Figure S5. Scanning electron microscopy images at different magnifications of (a) the raw residual olive skin itself and (b) the extracted solid obtained after microwave-assisted extraction at optimised conditions (100% ethanol, 99 °C for 4 min, and 10% *w*/*v* solid loading).
