# Supplementary material for: Lower Energy-Demanding Extraction of Bioactive Triterpene Acids by Microwave as the First Step towards Biorefining Residual Olive Skin
Source: Antioxidants (Basel). 2024 Oct 9;13(10):1212. doi: 10.3390/antiox13101212 (PMC11504040; doi:10.3390/antiox13101212)
Supplement: Supplementary file 1 [file antioxidants-13-01212-s001.zip › antioxidants-3219376-supplementary.pdf]

antioxidants

MDPI

Article

Lower Energy Demanding Extraction of Triterpene Acids by Microwave as the First Step towards Biorefining Residual Olive Skin

Irene Gómez-Cruz [1,2], María del Mar Contreras [1,2,*], Inmaculada Romero [1,2] and Eulogio Castro [1,2]

[1] Department of Chemical, Environmental and Materials Engineering, Universidad de Jaén, Campus Las Lagunillas, 23071 Jaén, Spain; igcruz@ujaen.es (I.G.-C.); iromero@ujaen.es (I.R.); ecastro@ujaen.es (E.C.)

[2] Institute of Biorefineries Research (I3B), University of Jaén, Campus Las Lagunillas, 23071 Jaén, Spain.

* Correspondence: mcgamez@ujaen.es

Supplementary materials

Figures

Citation: Gómez-Cruz, I.; Contreras, M.d.M.; Romero, I.; Castro, E. Lower Energy Demanding Extraction of Triterpene Acids by Microwave as the First Step towards Biorefining Residual Olive Skin. *Antioxidants* **2024**, *13*, 1212. https://doi.org/10.3390/antiox13101212

Academic Editor: Dimitrios Stagos

Received: 5 September 2024
Revised: 27 September 2024
Accepted: 3 October 2024
Published: 9 October 2024

Copyright: © 2024 by the authors. Licensee MDPI, Basel, Switzerland. This article is an open access article distributed under the terms and conditions of the Creative Commons Attribution (CC BY) license (https://creativecommons.org/licenses/by/4.0/).

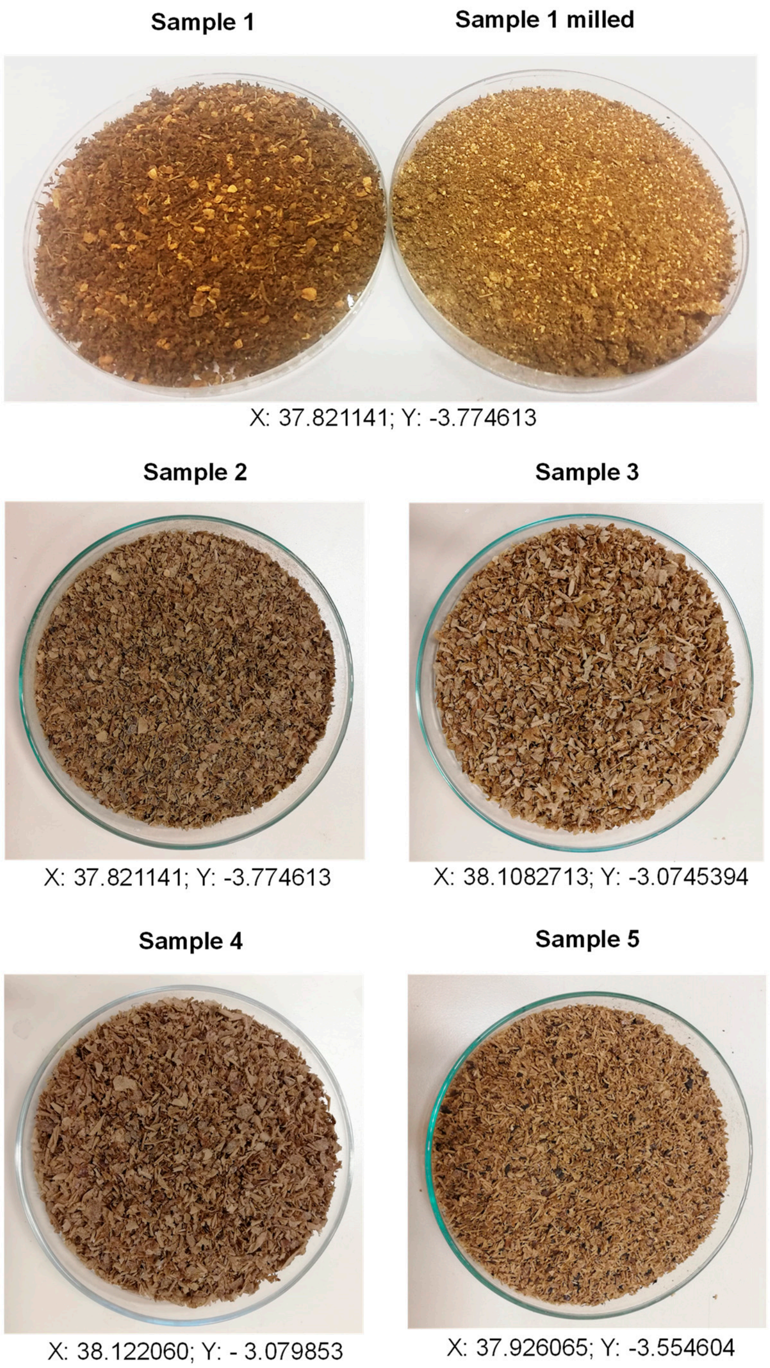

Figure S1. Images of the different residual olive skin samples and geographic coordinates of the local industries.

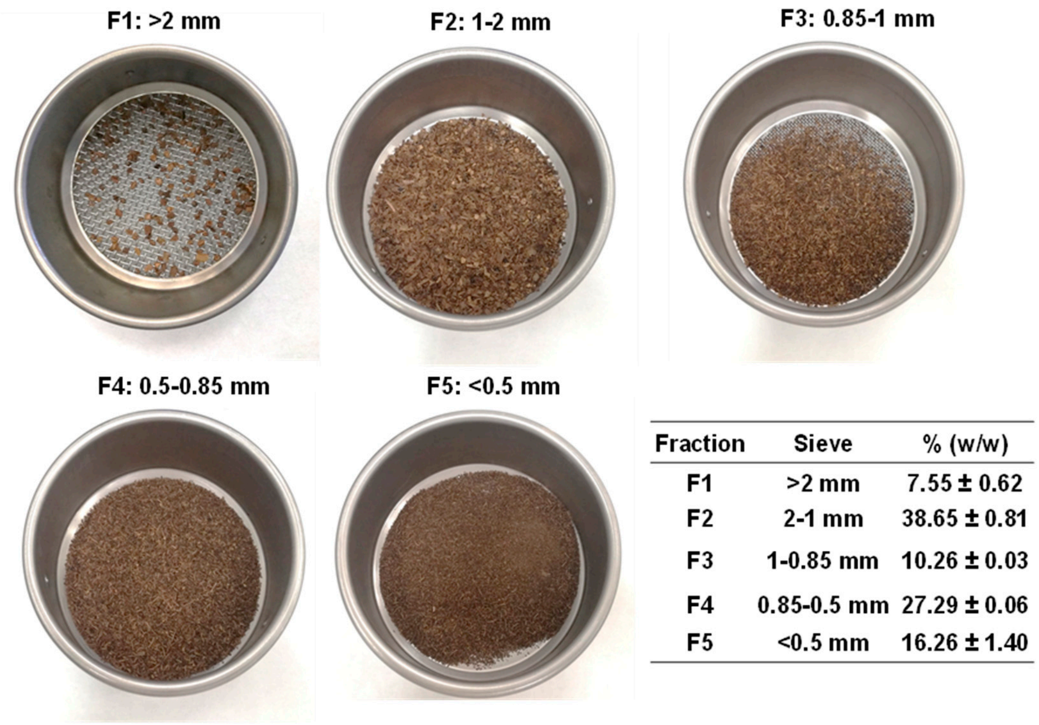

Fraction	Sieve	% (w/w)
F1	>2 mm	7.55 ± 0.62
F2	2-1 mm	38.65 ± 0.81
F3	1-0.85 mm	10.26 ± 0.03
F4	0.85-0.5 mm	27.29 ± 0.06
F5	<0.5 mm	16.26 ± 1.40

Figure S2. Images of residual olive skin after sieving and weight distribution.

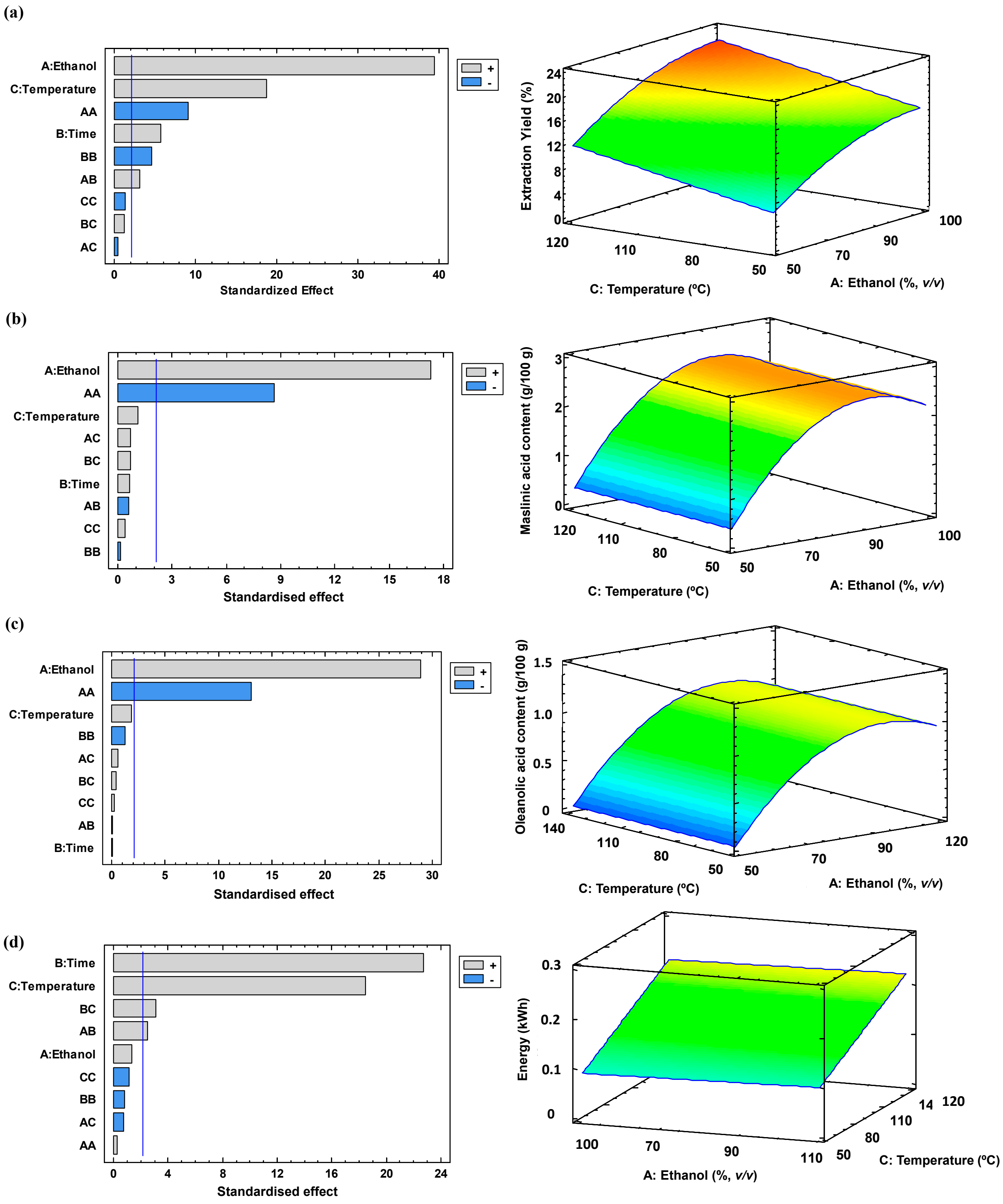

Figure S3. Effect of the independent factors in the response variables represented in Pareto charts (left) and response surface charts (right) as a function of ethanol percentage and extraction temperature: (**a**) extraction yield (%), (**b**) maslinic acid content (g/100 g), (**c**) oleanolic acid content (g/100 g) and, (**d**) energy consumption (kWh) for the milled residual olive skin. The holding time was fixed at 17 min.

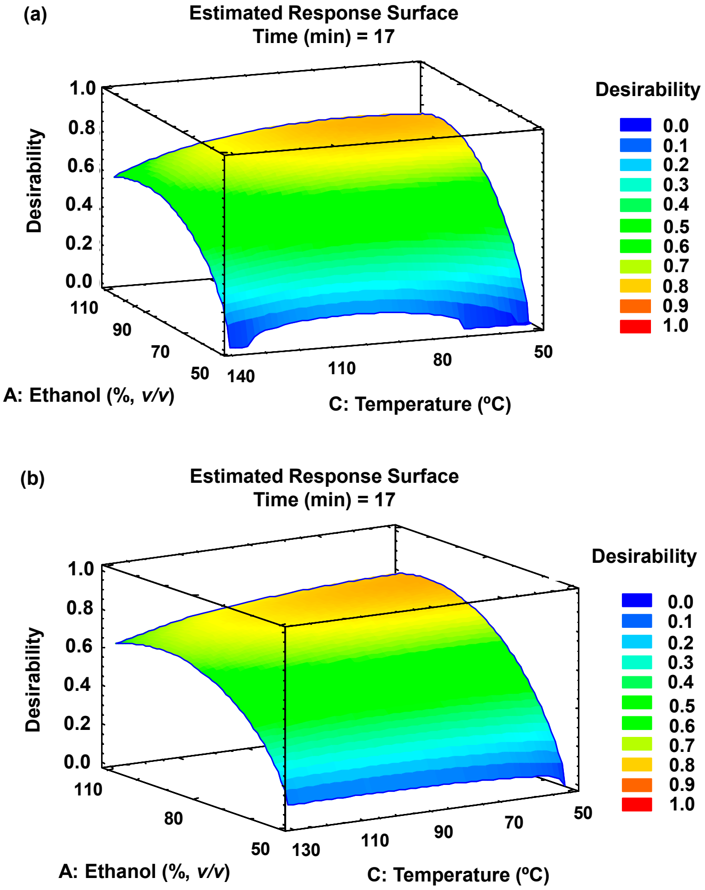

Figure S4. Three-dimensional plots of the overall desirability response surface for the effects of ethanol concentration and temperature in: (**a**) microwave-assisted extraction of the raw (unmilled) and (**b**) milled residual olive skin. The time was fixed at 17 min.

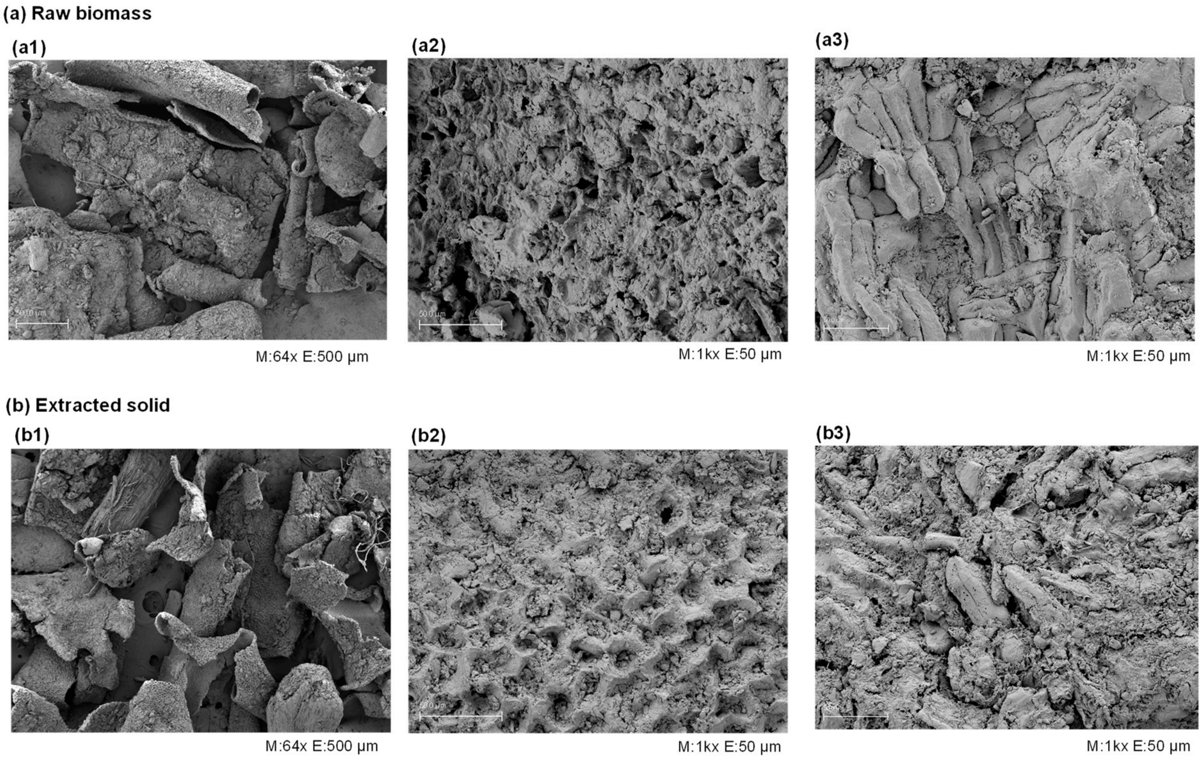

Figure S5. Scanning electron microscopy images at different magnifications of (**a**) the raw residual olive skin itself and (**b**) the extracted solid obtained after microwave-assisted extraction at optimised conditions (100% ethanol, 99 °C for 4 min, and 10% w/v solid loading).
